# The Hypothetical Psychological Impact of the COVID-19 Pandemic on Pediatrics and Pediatric Emergency Admissions: Evidence from Autoregressive Distributed Lag Model Method

**DOI:** 10.5152/eurasianjmed.2023.0165

**Published:** 2023-06-01

**Authors:** Hatice Genç Kavas, Ahmet Şengönül

**Affiliations:** 1 Department of Health Tourism Management, Sivas Cumhuriyet University, Social Sciences Institution, Sivas, Turkey; 2 Department of Econometrics, Sivas Cumhuriyet University, Faculty of Economics and Administrative Sciences, Sivas, Turkey

**Keywords:** Pediatry, psychiatry, covid-19, healthcare demand, time series analysis, patient behavior

## Abstract

**Objective:**

The COVID-19 pandemic and related deaths affected the number of admissions of patients to hospitals. However, no study has been found that examines the short and long-term psychological effects of children or their possible psychiatric admissions to hospitals during the pandemic period. In this context, the study aims to analyze the behaviors of individuals under the age of 18 in their health service utilization during the COVID-19 pandemic period.

**Materials and Method:**

For the study, whether the pandemic and psychiatry department (PSY) admissions affect the pediatrics department (PD) and pediatric emergency department (PED) admissions of children was investigated. The sample was taken from hospitals in Sivas between 2019 and 2021. Autoregressive distributed lag (ARDL) model is applied. The ARDL is an econometric method that can estimate the existence of the long-term correlations (cointegration) of variables and the short and long-term effects of explanatory variables on the dependent variable.

**Results:**

In the PED application model, the number of deaths, representing the impact of the pandemic, decreased the number of PED applications, while the number of vaccinations increased. On the other hand, applications to the PSY decreased in the short term, but increased in the long term. In the model of pediatric department admissions, in the long term, the number of new COVID-19 cases has decreased the number of PD admissions, while the number of vaccines has increased. While applications made to PSY in the short term have decreased the applications of PD, they have increased in the long term. As a result, the pandemic decreased both children's department admissions. In addition, admissions to PSY, which had greatly decreased in the short term, increased rapidly in the long term.

**Conclusion:**

Providing psychological support to both children and adolescents and their guardians during and after the pandemic should be included in planning.

**Main Points**

- The COVID-19 pandemic has caused dramatic decrease in hospital admissions of children and adolescents.
- While the number of daily new COVID-19 cases and new deaths in Turkey caused a decrease in hospital admissions, the vaccination rates caused an increase in hospital admissions, albeit very slightly.
- During COVID-19, the increase in patient admissions to psychiatry department (PSY) has decreased the number of admissions to PD and PED in the short term. In other words, in the short term, patients under the age of 18 may have preferred to apply to hospitals for psychological reasons over physiological reasons. In the long term, they may have applied to the PD and PED mostly for physiological reasons, with the end of the treatment process due to the increase in admissions to PSY.
- Especially the significant decrease in pediatric emergency visits may be evidence that these patients can be treated at home without going to the emergency departments, even in the absence of a pandemic.

## Introduction

On March 11, 2020, a worldwide pandemic was declared due to the new coronavirus disease (COVID-19/severe acute respiratory syndrome coronavirus 2), and as of the time of the study, more than 6 million people died from this disease. The rapid spread of the disease and the increase in cases show us that the threat continues globally. The instinct of survival is the main source of motivation for all living things. Especially in cases where the risk of death is visible, individuals may exhibit different behaviors in their daily routines to feed their motivation. The COVID-19 pandemic also has an impact on behavior as it causes an increasing number of deaths. During the pandemic, a full lockdown was experienced in the world in certain periods, but also in the periods when the closure was not mandatory, the behavior of people was outside of normal life behaviors. In this study, it was analyzed how the children and adolescents in Sivas behaved before and after the introduction of COVID-19 into our lives and during the vaccination process against the disease, when they applied to the hospital, and whether there were psychological effects based on their behavior. In the reviewed literature^1-3^ it was observed that while there was a significant increase in emergency department admissions for psychological reasons during COVID, there was a general decrease in admissions due to other diseases. And also, a study conducted in Turkey has shown that individuals are seriously affected psychologically by COVID-19.^4^ In this context, our study will hypothetically test whether there is a relationship between admissions to psychiatry departments and pediatrics departments, and pediatric emergency departments admissions. 

There are many studies on the impact of the COVID-19 pandemic on healthcare utilization. It is questioned the whereabouts of children who are supposed to be admitted to the PED in Manchester UK under normal circumstances and why they do not use healthcare services.^5^ The COVID-19 pandemic has had a major impact on non-COVID-19-related healthcare use, largely due to social restraint measures and changes in people's healthcare-seeking behavior; however, they say that the utilization of healthcare services for mental health disorders is increasing.^1^ The admissions to PED for psychiatric reasons of individuals under the age of 18 increased compared to the pre-pandemic period during the pandemic outbreak of the pandemic,^2,3^ and it can be seen by the studies that the demands for healthcare services, that is, admissions to PED, and the number of hospitalizations decreased very significantly in various regions of the World.^6-9^ Again, based on this period, it was mentioned that there was a significant decrease in the demand for serious treatment, including cancer,^10^ not only in PED but also in a wide variety of health services^11-13^ in the healthcare system. According to a study published in 2021, the use of many different types of hospitals such as hospital visits, emergency admissions, diagnosis, and treatment decreased very seriously.^14^ However, there are studies showing that admissions are shifted to the online system rather than face-to-face interviews^15^ in these situations. It has been observed that there are delays in the care and treatment admissions of even cancer patients in Turkey, and therefore, there are significant changes in the utilization of healthcare services^16^. It is also stated that the pandemic causes increased anxiety and depression, and this is more common, especially among women in Turkey.^4^ In this context, it can be seen that the COVID-19 pandemic is a major factor in healthcare utilization. However, when the studies were examined, it was not found that causality analysis was performed with explanatory variables affecting the use of healthcare services. 

## Materials and Methods

In our study, the weekly admission numbers of patients under the age of 18 between September 2019 and July 2021 in 4 hospitals within the scope of secondary and tertiary healthcare institutions in Sivas were used. The archive data were obtained from the statistics unit of all secondary and tertiary healthcare facilities in Sivas. In this study, the number of child patients who applied to the PSY, PD, and PED was included in models to measure the psychological impact on children. The cross-tabulation method using Statistical Package for Social Science Statistics 25 (IBM Corp., Armonk, NY, USA) was used to combine and classify the data. ARDL models used for econometric analyses were created with E-views 10 (IHS Global Inc., 4521 Campus Drive, #336 Irvine, CA 92612-2621, USA). Ethics Committee Report numbered 60263016-050.06.04-E.482051 and the research permits were obtained. The study was carried out retrospectively using the number of patient applications received from the statistics department of the hospitals. None of the personal data of patients are used. Informed consent was not obtained from the patients as there was no clinical study that used any personal data. Since there was no animal study, the NIH guidelines were not needed. As data, the number of weekly new cases, vaccinations, and deaths from COVID-19 in Turkey are used. The hypothesis to be tested in the study is that COVID-19 cases, deaths caused by COVID-19, vaccination against COVID-19, and PSY admissions that may affect PED and PD admissions. To test this hypothesis, a time series econometric model was established and analyzed, and a model known as the ARDL developed by Pesaran et al^17^ was estimated for this analysis. While the ordinary least squares (OLS) method cannot distinguish between short term and long term, the ARDL model assumes that a time series of dependent variables is a linear function of the previous lag values itself and the current and previous lag values of the explanatory variables.^18^ The ARDL model decomposes the effect of an explanatory variable on the dependent variable as short-term and long-term effects. For example, indeed, a model's influencing variable may sometimes increase the affected variable in the short run while decreasing it in the long run. For example, while a drug cures a disease within the required dose period (in the short term), if it is taken as an overdose (long term), it may make the patient worse.

In this context, 2 different models were considered. The models and their equations are as follows;

- Model 1: Pediatric Emergency (ER) (weekly deaths, weekly vaccines, weekly new cases, psychiatry)

### General Model

*Pediatric ER_t_*_ =_ *β_0_ +* *β_1_ weekly deaths + β_2_ weekly vaccines + β_3_ weekly new cases + β_4_ psychiatry + u_t_*

### Autoregressive Distributed Lag (ARDL) Model 


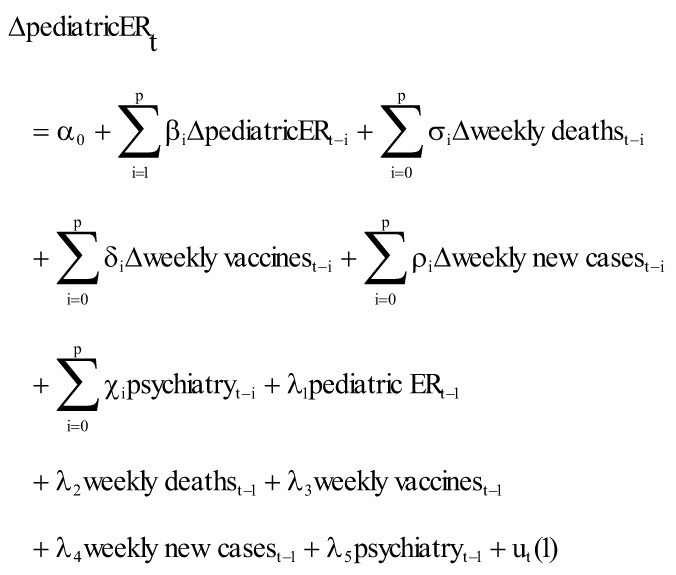

- Model 2: Pediatrics department (weekly deaths, weekly vaccines, weekly new cases, psychiatry)

### General Model

Pediatrics*_t_* = *β_0_ +* *β_1_ weekly deaths + β_2_ weekly vaccines + β_3_ weekly new cases + β_4_ psychiatry + u_t_*

### Autoregressive Distributed Lag (ARDL) Model


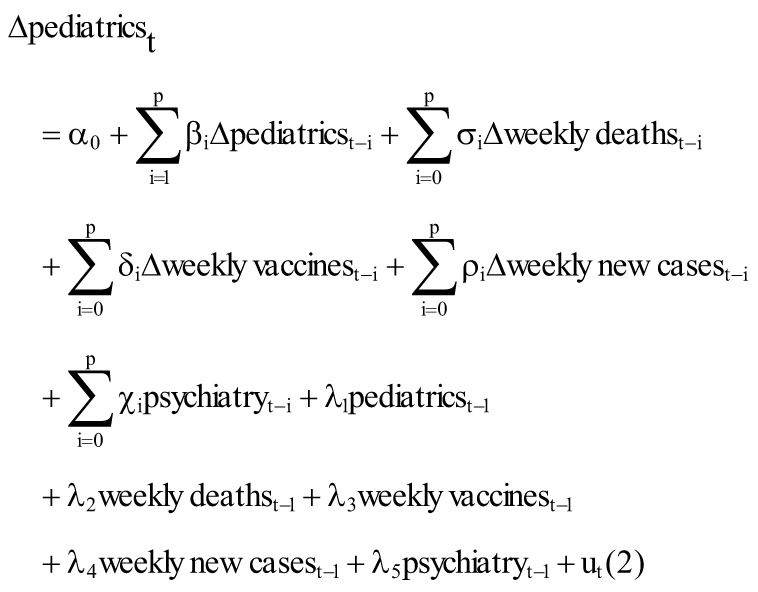


### Definitions

The data set used in the models consists of the number of applications made to 3 different departments in the hospitals, the number of weekly new COVID-19 cases, the weekly number of deaths from COVID-19, and the weekly vaccination numbers against COVID-19. The data were retrospectively collected.

The departments in the models are pediatric emergency departments (PED), pediatrics departments (PD), and psychiatry departments (PSY). 

*Pediatric emergency departments:* The number of applications made to PED represents PED in all the secondary and tertiary healthcare facilities in the city. All individuals under the age of 18 can apply to these departments in case of emergency or acute diseases.

*Pediatrics departments: *PD is a branch of science that deals with the diagnosis, follow-up, and treatment of individuals aged 0-17. In the study, the number of applications made to PD in all secondary and tertiary healthcare facilities in the city was used.

*Psychiatry department*: It is the service that individuals go to get psychiatric support. In the study, all applications made to psychiatry and psychology departments where individuals under the age of 18 applied for examination were evaluated as PSY admissions.

*New cases:* It is the number of new COVID-19 cases per week in Turkey.

*Deaths: *It is the number of deaths due to COVID-19 in Turkey per week.

*Vaccine: *It is the number of vaccinations against COVID-19 disease per week in Turkey.

**Figure 1.**
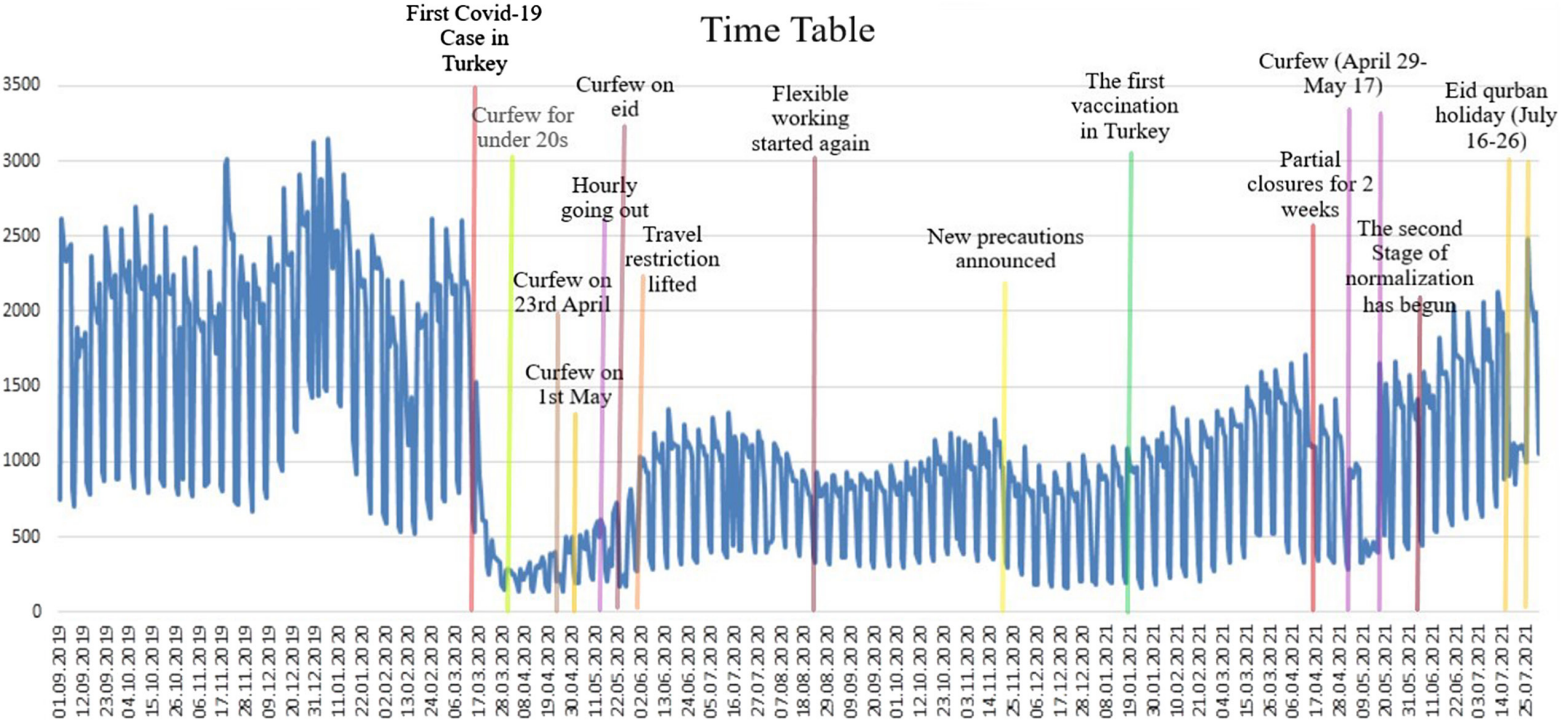
All admissions of individuals under 18s to secondary and tertiary healthcare facilities in Sivas between Semtember 1, 2019 and July 31, 2021. *Time indicators have been added to the chart based on the announcements of the relevant ministries.^19,20^ **Departments that require treatment such as cancer, oncology, departments associated with these departments, family medicine, dentistry departments, and Covid-19 admissions were not included.

*Holidays: *Refers to the national and religious holidays in Turkey during the time period within the scope of the study. Holidays, curfews, and restrictions are shown on the timeline in Figure 1.

## Results

Table 1 shows the admissions made to all secondary and tertiary healthcare facilities in Sivas between September 1, 2019, and July 31, 2021. 

The total number of admissions of children and adolescents to the PED, PD, and PSY of the secondary and tertiary healthcare facilities in Sivas during the current date range was 456.795. As the departments, PED (49.59% of the total admissions), PD (46.49% of the total admissions), and PSY (total of psychiatry and psychologist departments−3.91% of the total admissions) were taken as variables. 

Figure 1 shows the graph of the number of children and adolescents who applied to the hospitals within the scope of the study between September 1, 2019, and July 31, 2021, and the timeline of cases, vaccinations, closures, and holidays in Turkey. The first COVID-19 case was seen in Turkey on March 11, 2020. The curfew for individuals under the 20s was declared on April 3, 2020. Mass vaccination started on January 14. The first COVID-19 case in Turkey and the declaration of the disease as a pandemic occurred simultaneously, and after this date, hospital admissions of children and adolescents in Sivas decreased dramatically. During the religious and national holidays before and after the onset of the pandemic and curfews, utilization of healthcare was minimal as the doctors of outpatient clinics were not working at that time in the outpatient clinics. 

**Figure 2.**
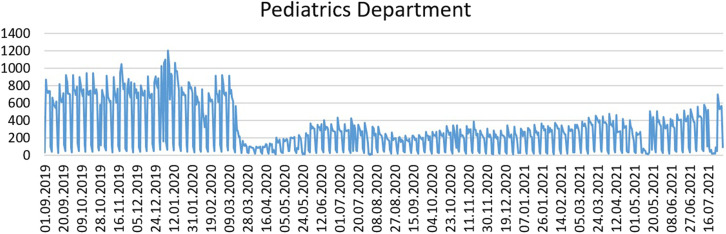
Application of individuals under 18 to secondary and tertiary healthcare facilities’ pediatrics departments in Sivas between January 09, 2019 and December 07, 2021.

Admissions of individuals to PD decreased significantly with the first COVID-19 case in Turkey (Figure 2). During the pandemic and even after vaccinations, admissions remained well below pre-pandemic levels. 

**Figure 3.**
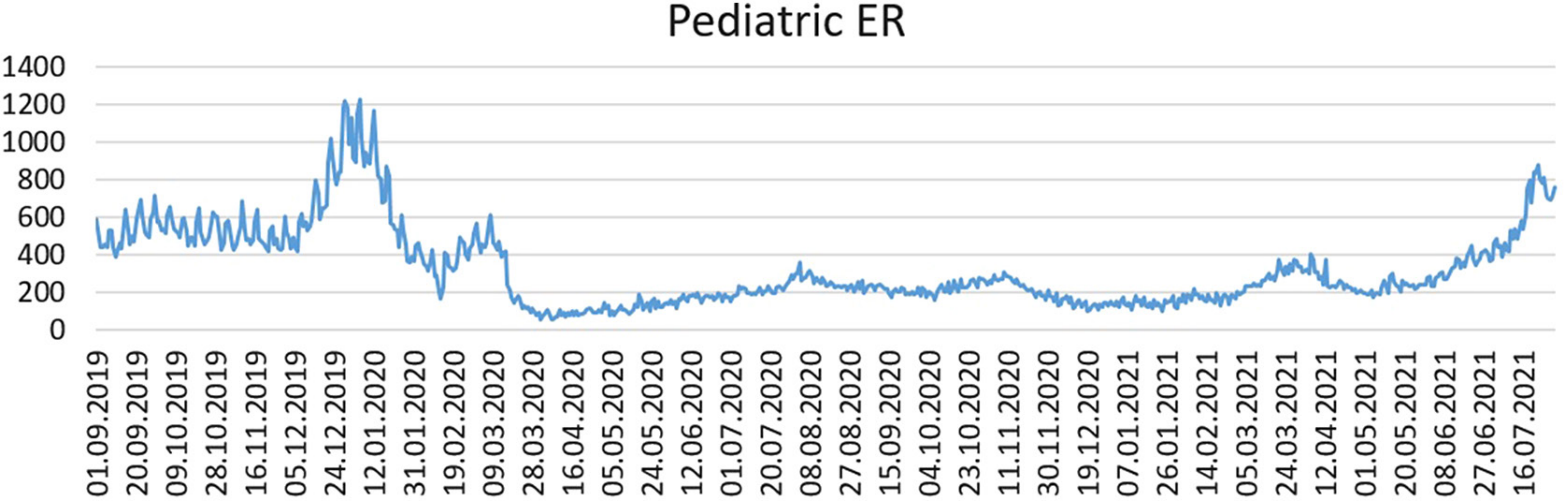
Application of individuals under 18s to secondary and tertiary healthcare facilities’ pediatric emergency departments in Sivas between September 1, 2019 and July 31, 2021.

As seen in Figure 3, with the first occurrence of the COVID-19 case in Turkey, the number of PED admissions in Sivas decreased significantly. In July 2021, admissions to PED tended to return to pre-pandemic values.

**Figure 4.**
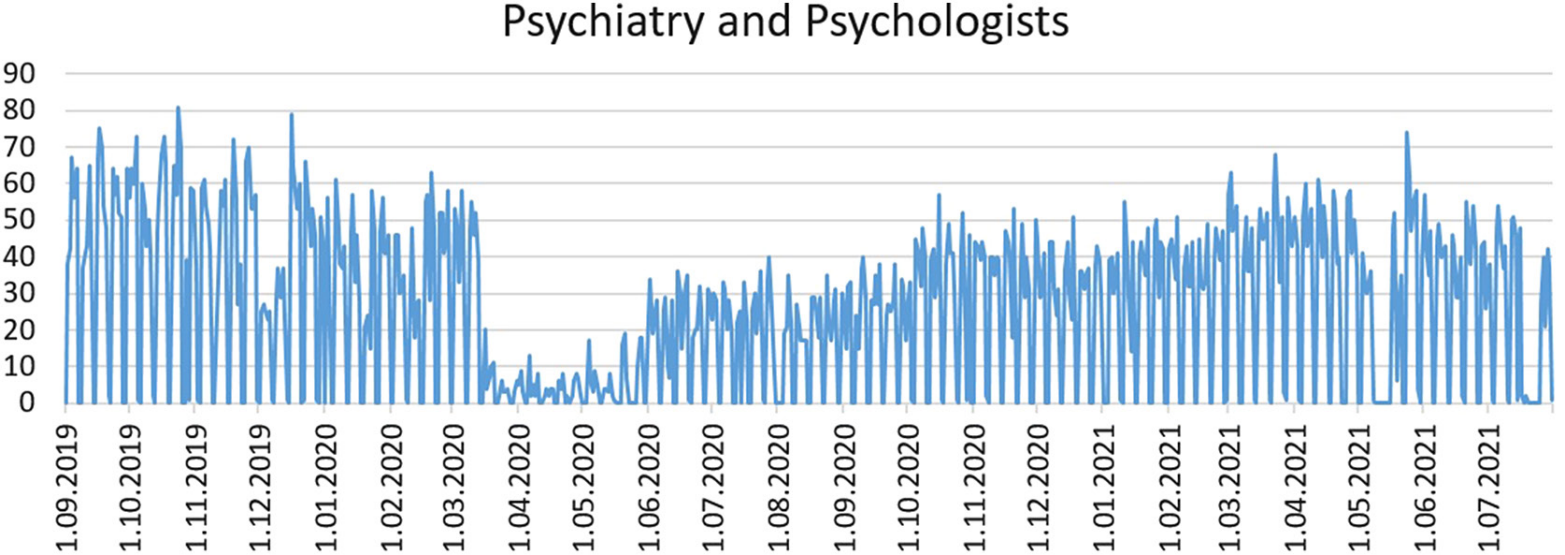
Application of Individuals Under 18s to Secondary and Tertiary Healthcare Facilities’ Psychiatry and Psychologist Departments in Sivas between September 1, 2019 and July 31, 2021.

Admissions to PSY of children and adolescents decreased with COVID-19 (Figure 4). 

According to the models discussed, the dependent variables are the number of patients in the relevant department (PED — Model 1 and PD — Model 2) who applied to all secondary and tertiary healthcare hospitals in Sivas to use health services pre-COVID-19 and post-COVID-19 (September 1, 2019 to July 31, 2021). The explanatory variables of the models are the number of deaths per week from COVID-19, the number of vaccination per week, the number of new cases per week, as well as the number of weekly psychiatry admissions for model 1 and model 2.

### Model 1: Pediatric ER (Weekly Deaths, Weekly Vaccines, Weekly New Cases, Psychiatry)

As can be seen in Table 2, since the adjusted R^2^ is 0.95 in the ARDL equation, explanatory variables explain the model as a whole at a rate of 95%. According to ARDL results, a long-term causal relationship was found between PED admissions and psychiatry admissions, the number of deaths, and the number of vaccinations. While admissions to PSY and the number of daily vaccinations increase the number of applications to PED, the number of deaths decreases. Considering the short-term relationships, the number of vaccinations in the last 12 weeks, excluding the number of vaccinations 5 weeks ago (*P* = .2133), decreased the number of admissions to PED (e.g., 10.000 vaccinations 6 weeks ago decreases the number of PED visits this week by 3 — coefficient = −0.000347/*P* = .0038—). While death cases show their effect in the long term, there is no significant effect at the *P* = .05 level in the short term. While admissions to PSY increased the number of PED admissions in the long term, it decreased admissions with a very high effect in the short term (e.g., 1 PSY admission 7 weeks ago decreases the number of admissions to the PED this week by three — coefficient= −3.260418/*P* = .0060 —).

The number of admissions to the PED in the previous first, fifth, and eighth weeks increase PED admissions this week. (e.g., 10 admissions to PED 5 weeks ago increase the number of admissions to PED by 4 this week). 

### Model 2: Pediatrics Department (Weekly Deaths, Weekly Vaccines, Weekly New Cases, Psychiatry)

As can be seen in Table 3, since the adjusted R^2^ is 0.948 in the ARDL equation, the explanatory variables explain the model as a whole at a rate of approximately 95%. Again, according to the ARDL long-term equation, the number of admissions to PD is affected at the *P* = .05 level depending on all other explanatory variables, except for death cases. However, while PSY admissions (coefficient = 11.28882, *P* = .0000) and vaccination (coefficient = 0.001015, *P* = .0044) increase the admissions to PD, the number of daily cases (coefficient = −0.015408, *P* = . 0320) decreases. Considering the short-term co-integration, PD admissions are affected by all variables weekly, including the number of weekly deaths. While PSY admissions, death, and vaccination decrease the number of admissions to PD in the short term, the number of daily cases and admissions to PD has an increasing effect (e.g., each death 3 weeks ago decreases the number of pediatrics admissions this week by 2). Around 10 000 vaccines administered 1 week ago in Turkey decreases the number of applications made to PD by 6 this week. One PSY admission 7 weeks ago decreases PD admissions by 10 this week and 100 weekly cases of COVID-19 that emerged 4 weeks ago increased PD admissions by 1 this week, and 1 admission to PD 8 weeks ago increases by 1 admission to PD this week). 

## Discussion

In this study, the changes in the admissions of individuals under the age of 18 to PD and PED of the secondary and tertiary healthcare facilities in Sivas city center and how their motivations for these changes were affected in the short and long term were examined. According to econometric models; dependent variables in each model are explained very highly by explanatory variables (R^2^_Model1_ = 0.95 and R^2^_Model2_ = 0.95,). With the introduction of the COVID-19 disease into our lives, it can be understood from the time series analyzed that there has been a significant decrease in the admissions of individuals under the age of 18s to hospitals in Sivas. To explain the reason for the decrease in the number of PD and PED admissions, weekly deaths, weekly vaccinations, and weekly cases were added to the models, but a significant causal relationship could not be found. For this reason, in addition to these explanatory variables, other departments’ admissions were added to the models. Among the explanatory variables made to PD and PED admissions models, the highest causality relationship was obtained by adding the admissions to PSY to the models. As a result, econometric models were analyzed based on the admissions to PSY. In both Models, co-integration (long-short term) relationships were found at the 1% significance level (Model 1’s explanatory variables in long term; deaths (coefficient = −1.97; *P* = .0001), vaccines (coefficient = 0.0005; *P* = .0303), psychiatry (coefficient = 15.78; *P* = .0000); Model 2’s explanatory variables in long term; vaccines (coefficient = 0.0010; *P* = .0044), new cases (coefficient = −0.015; *P* = .0320), psychiatry (coefficient = 11.288; *P* = .0000). Co-integration–causality relationships in short-term equations be seen in Table 2 and 3. According to the results obtained, it has been revealed that the admissions of individuals under the age of 18 in Sivas to PED are mostly affected by death cases in the long and short term, while the admissions to PD are affected by death cases in the short term. In this context, it can be said that the psychological effect of death has become more specific with COVID-19. Considering the ARDL results in Table 2, long-term admissions of children and adolescents to PSY have an increasing effect on PED admissions (1 PSY admission increases PED admissions by 15). Considering the short term, PSY admissions decrease the number of admissions to PED. According to the ARDL results in Table 3, while admissions to PSY have an increasing effect on PD admissions in the long term, it has a decreasing effect in the short term. ARDL results show us that there is an important correlation between PSY, PD, and PED visits. For PSY admissions (Figure 4), an increasing trend is observed in a short time unlike in other departments. It even reached the pre-pandemic period values about 1 year later. Admissions to the hospital for mental health were different from those for physical illnesses. The proportion of individuals under the age of 18 who applied to PSY during the COVID-19 period we studied was not significantly affected as in other departments. The need for psychological support during the pandemic period has not disappeared in any way, on the contrary, it has increased as time progressed. Besides, according to our econometric analysis, it can be seen that psychology may affect physiology and mental health may be more prominent than physical disorders during the pandemic period. Because, while it is seen that psychological relief from PSY visits decreases PD and PED admissions in the short term, it can be interpreted that this relief may cause an increase again in the long term when this relief ends. In addition, the healthcare demand of children and adolescents should not be considered separately from their parents or guardians. In this context, it can be interpreted that individuals under the age of 18 who apply to PED and those who are responsible for them experience short-term relief, but in the long term, individuals need psychological support (Table 2). The number of new COVID-19 cases, the number of deaths, and the number of vaccinations added to the models may be an indicator of psychological behavior. Because when the PSY admissions were examined, a very high correlation was obtained. In the literature, there are studies on the increase in hospital and emergency department visits due to mental illnesses (such as suicide, depression, and anxiety) along with the pandemic.^1-3^ It was seen that individuals were psychologically affected by the pandemic, especially women in Turkey.^4^ Considering the effects of PSY admissions in PD and PED admissions in models, and the increase in mental illnesses during the pandemic period, it can be interpreted that mental ailments can trigger physical ailments. Providing psychological support to both children and adolescents and their guardians during pandemic periods will ensure the growth of healthier individuals. When all models are evaluated, thinking about the existence of psychological effects in healthcare utilisations will lead to correct solutions. Apart from these, considering all admissions to hospitals, the COVID-19 pandemic may have caused individuals to avoid treatment for diseases other than COVID-19 that may cause them to lose their health. Although patients from both other provinces and abroad came to the hospitals in Sivas included in the study, the inability to make a generalization for the use of health services in other provinces may be the limitation of this study. At the same time, only secondary and tertiary healthcare facilities admissions were included in the study, and primary healthcare facilities were excluded. The lack of a case-based analysis is the shortcoming of the study. In the literature, there are studies on the demand for health services for children and adolescents during the COVID-19 period. However, as in our study, a detailed examination of the causality relationship between departments has not been analyzed as far as we know. In addition, such a study does not exist in Turkey as far as we know. We can say that our study offers unique information for the pandemic and post-pandemic periods. For this reason, it will be a basis for longer-term and many comprehensive kinds of research to be studied in the future. Our study, which we handle with an interdisciplinary approach, will make important contributions to many fields such as health economics, health services planning, patient behavior, marketing, supply and demand, insurance, sociology, and psychology.

## Figures and Tables

**Table 1.** Statistics of Patients Under the Age of 18 Applying to the Secondary and Tertiary Healthcare Hospitals in Sivas between September 1, 2019, and July 31, 2021

		N	%
Department	Pediatric ER	226.527	49.59
	Pediatric Department	212.390	46.49
	Psychiatry	17.878	3.91
	Total	456.795	100.00

**Table 2.** Co-integration and ARDL Results for Pediatric Emergency Department

Dependent Variable: Pediatric ER		Observation Period: 2019-2021		
Test Name: ARDL		Selected Model: ARDL (9, 1, 12, 1, 12)		
**Variable**	**Coefficient**	**Standard Error**	***t*****-Statistic**	***P***
Long-term equation				
Death	−1.971082	0.463309	−4.254361	.0001
Vaccine	0.000538	0.000241	2.232478	.0303
New cases	0.006747	0.004519	1.492900	.1420
Psychiatry	15.78170	1.783727	8.847599	.0000
C	222.7474	328.8044	0.677447	.5014
Short-term equation				
D(Pediatric ER(−1))	0.868724	0.121068	7.175499	.0000
D(Pediatric ER (−2))	0.195560	0.149629	1.306973	.1974
D(Pediatric ER (−3))	0.115621	0.138299	0.836021	.4073
D(Pediatric ER (−4))	−0.016637	0.131373	−0.126640	.8998
D(Pediatric ER (−5))	0.401495	0.125440	3.200704	.0024
D(Pediatric ER (−6))	−0.207057	0.134804	−1.535984	.1311
D(Pediatric ER (−7))	0.213938	0.134362	1.592251	.1179
D(Pediatric ER (−8))	0.407690	0.136303	2.991054	.0044
D(Death)	−0.021157	0.336300	−0.062910	.9501
D(Vaccine)	−0.000159	6.92E-05	−2.302391	.0257
D(Vaccine (−1))	−0.000374	0.000102	−3.666.121	.0006
D(Vaccine (−2))	−0.000356	0.000102	−3.503601	.0010
D(Vaccine (−3))	−0.000524	0.000108	−4.835854	.0000
D(Vaccine (−4))	−0.000238	0.000101	−2.360898	.0223
D(Vaccine (−5))	−0.000135	0.000107	−1.261252	.2133
D(Vaccine (−6))	−0.000347	0.000114	−3.041973	.0038
D(Vaccine (−7))	−0.000472	0.000158	−2.984513	.0045
D(Vaccine (−8))	−0.000847	0.000184	−4.596964	.0000
D(Vaccine (−9))	−0.000792	0.000215	−3.692346	.0006
D(Vaccine (−10))	−0.000775	0.000211	−3.673309	.0006
D(Vaccine (−11))	−0.000526	0.000175	−3.004671	.0042
D(New Cases)	−9.98E-05	0.001693	−0.058927	.9533
D(Psychiatry)	0.816681	0.899329	0.908101	.3684
D(Psychiatry (−1))	−5.295801	1.376645	−3.846890	.0004
D(Psychiatry (−2))	−4.744132	1.315391	−3.606633	.0007
D(Psychiatry (−3))	−5.027811	1.286689	−3.907558	.0003
D(Psychiatry (−4))	−4.479287	1.109096	−4.038681	.0002
D(Psychiatry (−5))	−3.109667	1.118099	−2.781210	.0077
D(Psychiatry (−6))	−4.116569	1.164386	−3.535400	.0009
D(Psychiatry (−7))	−3.260418	1.132846	−2.878078	.0060
D(Psychiatry (−8))	−4.681898	1.144411	−4.091099	.0002
D(Psychiatry (−9))	−3.604724	1.100926	−3.274266	.0020
D(Psychiatry (−10))	−2.006856	0.961393	−2.087446	.0422
D(Psychiatry (−11))	−1.704754	0.845258	−2.016845	.0493
CointEq(−1)*	−0.395261	0.069452	−5.691139	.0000
	F-Statistic	Signif icance	I(0)	I(1)
Bounds test	4.888916	1%	3.29	4.37
R^2^	0.975349			
Adjusted R^2^	0.955321			

ARDL, autoregressive distributed lag model.

**Table 3.** Co-Integration and ARDL Results for Pediatrics Department

Dependent Variable: Pediatrics		Observation Period: 2019-2021		
Test Name: ARDL		Selected Model: ARDL (12, 6, 12, 10, 11)		
**Variable**	**Coefficient**	**Standard Error**	***t*****-Statistic**	***Prob.***
**Long-term equation**				
**Death**	−0.219124	0.643104	−0.340728	.7355
**Vaccine**	0.001015	0.000331	3.063576	.0044
**New cases**	−0.015408	0.006873	−2.241916	.0320
**Psychiatry**	11.28882	1.117283	1.010381	.0000
**C**	680.8998	2.126355	3.202193	.0031
Short Term Equation				
**D(Pediatrics(−1))**	0.136120	0.135507	1.004526	.3227
**D(Pediatrics(−2))**	0.011128	0.129335	0.086042	.9320
**D(Pediatrics(−3))**	0.039347	0.118067	0.333255	.7411
**D(Pediatrics(−4))**	0.052735	0.108438	0.486316	.6301
**D(Pediatrics(−5))**	0.321142	0.111408	2.882580	.0070
**D(Pediatrics(−6))**	0.597210	0.131479	4.542253	.0001
**D(Pediatrics(−7))**	0.703773	0.156473	4.497734	.0001
**D(Pediatrics(−8))**	1.134601	0.169973	6.675188	.0000
**D(Pediatrics(−9))**	0.924576	0.190114	4.863269	.0000
**D(Pediatrics(−10))**	0.521924	0.162577	3.210312	.0030
**D(Pediatrics(−11))**	0.200468	0.115057	1.742342	.0911
**D(Death)**	−0.968932	0.516655	−1.875394	.0699
**D(Death(−1))**	−1.624827	0.540556	−3.005844	.0051
**D(Death(−2))**	−0.760088	0.497880	−1.526650	.1367
**D(Death(−3))**	−2.124096	0.533681	−3.980089	.0004
**D(Death(−4))**	0.762737	0.544337	1.401221	.1708
**D(Death(−5))**	−1.356942	0.547963	−2.476336	.0187
**D(Vaccine)**	5.28E-05	8.12E-05	0.649723	.5205
**D(Vaccine(−1))**	−0.000697	0.000141	−4.927075	.0000
**D(Vaccine(−2))**	−0.000721	0.000142	−5.064209	.0000
**D(Vaccine(−3))**	−0.000720	0.000162	−4.431073	.0001
**D(Vaccine(−4))**	−0.000854	0.000176	−4.866121	.0000
**D(Vaccine(−5))**	−0.000889	0.000174	−5.116743	.0000
**D(Vaccine(−6))**	−0.000757	0.000197	−3.832990	.0006
**D(Vaccine(−7))**	−0.001076	0.000206	−5.232050	.0000
**D(Vaccine(−8))**	−0.000646	0.000196	−3.294723	.0024
**D(Vaccine(−9))**	−0.000735	0.000196	−3.744446	.0007
**D(New cases)**	0.002189	0.002111	1.037245	.3074
**D(New cases(−1))**	0.013324	0.003054	4.363197	.0001
**D(New cases(−2))**	0.016010	0.003180	5.034282	.0000
**D(New cases(−3))**	0.011588	0.003374	3.434670	.0017
**D New cases(−4))**	0.013454	0.003117	4.316633	.0001
**D(New cases(−5))**	0.012502	0.003134	3.988975	.0004
**D(New cases(−6))**	0.010879	0.002621	4.150736	.0002
**D(New cases(−7))**	0.006034	0.002700	2.235168	.0325
**D New cases(−8))**	0.008324	0.002453	3.393161	.0019
**D(New cases(−9))**	0.003598	0.002756	1.305484	.2010
**D(New cases(−10))**	0.005963	0.002153	2.769691	.0093
**D(Psychiatry)**	3.471791	0.850263	4.083195	.0003
**D(Psychiatry(−1))**	−7.355510	1.950862	−3.770390	.0007
**D(Psychiatry(−2))**	−5.275913	2.019113	−2.612985	.0136
**D(Psychiatry(−3))**	−6.961026	1.836116	−3.791169	.0006
**D(Psychiatry(−4))**	−5.615754	1.648729	−3.406110	.0018
**D(Psychiatry(−5))**	−6.975836	1.532279	−4.552588	.0001
**D(Psychiatry(−6))**	−8.419048	1.564537	−5.381176	.0000
**D(Psychiatry(−7))**	−1.030237	1.941541	−5.306285	.0000
**D(Psychiatry(−8))**	−1.515088	2.134547	−7.097935	.0000
**D(Psychiatry(−9))**	−1.217584	2.510341	−4.850274	.0000
**D(Psychiatry(−10))**	−8.420010	2.046209	−4.114931	.0003
**D(Psychiatry(−11))**	−4.152366	1.428880	−2.906028	.0066
CointEq(−1)*	−0.684943	0.119356	−5.738653	.0000
	**F-Statistic**	**Significance**	**I(0)**	**I(1)**
**Bounds test**	**4.746975**	**1%**	**3.29**	**4.37**
**R2**	**0.981050**			
**Adjusted R2**	**0.948480**			

ARDL, autoregressive distributed lag model.
